# Human Immunodeficiency Virus Playing Hide-and-Seek: Understanding the T_FH_ Cell Reservoir and Proposing Strategies to Overcome the Follicle Sanctuary

**DOI:** 10.3389/fimmu.2017.00622

**Published:** 2017-05-31

**Authors:** Yew Ann Leong, Anurag Atnerkar, Di Yu

**Affiliations:** ^1^Infection and Immunity Program, Department of Biochemistry and Molecular Biology, Monash Biomedicine Discovery Institute, Monash University, Clayton, VIC, Australia; ^2^Department of Immunology and Infectious Disease, John Curtin School of Medical Research, The Australian National University, Canberra, ACT, Australia

**Keywords:** human immunodeficiency virus reservoir, cytotoxic T lymphocytes, follicular helper T cells, T_FH_ reservoir, B cell follicle sanctuary

## Abstract

Human immunodeficiency virus (HIV) infects millions of people worldwide, and new cases continue to emerge. Once infected, the virus cannot be cleared by the immune system and causes acquired immunodeficiency syndrome. Combination antiretroviral therapeutic regimen effectively suppresses viral replication and halts disease progression. The treatment, however, does not eliminate the virus-infected cells, and interruption of treatment inevitably leads to viral rebound. The rebound virus originates from a group of virus-infected cells referred to as the cellular reservoir of HIV. Identifying and eliminating the HIV reservoir will prevent viral rebound and cure HIV infection. In this review, we focus on a recently discovered HIV reservoir in a subset of CD4^+^ T cells called the follicular helper T (T_FH_) cells. We describe the potential mechanisms for the emergence of reservoir in T_FH_ cells, and the strategies to target and eliminate this viral reservoir.

## Overview

Chronic infection caused by human immunodeficiency virus (HIV) is a global epidemic that leads to lifelong diseases and imposes significant health burdens (1). HIV targets the immune system by inducing the death of CD4^+^ T helper cells, a critical cellular component of the adaptive immune system. The death of CD4^+^ T cells cripples the immune system and increases the susceptibility of the host to opportunistic infections—a condition known as acquired immunodeficiency syndrome (AIDS). Combination antiretroviral therapeutic regimen (cART) effectively inhibits the life cycle of the virus to prevent viral dissemination in the body (2–4). Nevertheless, despite the effective suppression of viremia by cART, the underlying inflammation and dysfunctional immune response continue to pose significant health challenges to HIV-infected individuals (5–8). Most importantly, the ongoing administration of drugs is required; otherwise, the interruption of therapy inevitably leads to viral rebound and progression to AIDS (9–11). Viral rebound originates from cells harboring HIV that escape eradication by cART. These cells form the HIV viral reservoir (12). Identifying the reservoir and devising effective strategies for its elimination are the keys to realizing the goal of curing HIV. Follicular T helper (T_FH_) cells are a specialized subset of CD4^+^ T cells that reside in B cell follicles to assist the humoral immune response (13). Recently, T_FH_ cells have been identified as the major cellular reservoir of HIV among CD4^+^ T helper cells (14–25). In this review, we focus on understanding the emergence of the T_FH_ reservoir and propose strategies to target and eliminate the reservoir.

### HIV Life Cycle and Stages of Disease

Human immunodeficiency virus is a single-stranded enveloped RNA virus that utilizes CD4 as the primary receptor for viral entry into the cells (26). HIV further subdivides to CC chemokine receptor 5 (CCR5) and CXC chemokine receptor-4 (CXCR4) tropic strains, which recognize the corresponding molecules on host cells as co-receptors for viral entry (27). As CD4 is the primary receptor for viral entry, the major target cells for productive HIV infection are CD4^+^ T cells (28, 29) and, to a lesser extent, macrophages (30). After viral entry, HIV releases a single-stranded RNA genome into the host cytoplasm. The RNA is synthesized into double-stranded complementary DNA (cDNA) by the viral reverse transcriptase. The cDNA is imported into the nucleus and integrated into host chromatin *via* viral integrase. The integrated cDNA—the provirus—is transcribed to produce viral RNA and proteins to form new virus to infect other cells (2). After HIV infection, viremia increases, with concomitant depletion of CD4^+^ T cells (31). The peak of viremia coincides with the activation of an anti-HIV immune response that leads to a brief reduction of viremia, which accompanies a transient recovery in the number of CD4^+^ T cells. This phase is the acute stage of the infection. The transient recovery of CD4^+^ T cells is then followed by their gradual depletion and a progressive increase of viremia, which constitute the chronic phase of the infection (31). If the infection is left untreated, the number of CD4^+^ T cells eventually falls below a critical level and the immunocompromised patient may die from AIDS-related complications (31). The changes in the number of CD4^+^ T cells are believed to be caused by virally induced direct or indirect cytopathic effect, which is mediated by both caspase-dependent and caspase-independent pathways (32–34). Cytotoxic CD8^+^ T lymphocytes (CTLs) are also implicated in the control of viremia and the death of infected CD4^+^ T cells (35, 36), and are described in more detail below.

### cART and Disease Controllers

The administration of cART suppresses plasma viremia to an undetectable level in a majority of HIV-infected patients (2). A typical cART uses small molecule inhibitors that target different components of the virus replication cycle, such as reverse transcriptase, viral protease, and integrase, while additional drugs can be employed to target host components such as the co-receptor for viral entry, CCR5 (2). Nevertheless, cART is unable to remove the provirus that has been integrated into the host genome. This is the major limitation of cART: even after the successful suppression of plasma viremia, new virus can be regenerated from the integrated provirus when treatment is interrupted. These cells together form the HIV cellular reservoir (12). Therefore, novel therapies that target and eliminate the viral reservoir are needed to prevent viral rebound from those cells—that is, a cure for HIV [reviewed by Katlama et al. (37)].

There are two strategies for the cure of HIV: the sterilizing cure and functional cure (37). The sterilizing cure involves the removal from the body of every integrated provirus that is able to spawn virus, while the functional cure aims to suppress viral rebound using the body’s immune system without the complete removal of provirus (37).

So far, the only case of a sterilizing cure is referred to as the “Berlin patient” case. In that case, an HIV-infected patient who suffered acute myelogenous leukemia received myeloablative chemotherapy and irradiation, which was followed by the transplantation of bone marrow cells from a CCR5Δ32 donor (38, 39). CCR5Δ32 is a deleterious mutation that abrogates CCR5 expression on the cell surface (38, 39). cART was discontinued after engraftment of the CCR5Δ32 bone marrow cells, and viral rebound has not yet been observed 8 years after the procedures, implicating a sterilizing cure of HIV. Although this case renewed interest in the search for a sterilizing cure, this method would be invasive to an otherwise healthy patient and expensive to implement on a larger scale.

However, a functional cure has occurred “naturally” in a few individuals (<5% of those infected) who have the ability to spontaneously suppress viremia without antiretroviral therapy (40). These patients are referred to as “elite controllers” or “long-term non-progressors” (40). They possess protective HLA haplotypes and potent anti-HIV CTL responses, which may contribute to their smaller viral reservoirs compared with disease progressors (35, 36). Some other patients (<1% of cART-treated), known as post-interruption viremia controllers (PIVCs) (41), are able to spontaneously suppress virus after treatment is interrupted. Interestingly, PIVC patients are not distinguished by a protective HLA subtype: their recovery is correlated more with their low viral load at the time cART is commenced (42, 43) and with the very early initiation of cART (41). Understanding the mechanisms of viral suppression in these individuals will provide important insight that may enable functional cure in disease progressors.

### Viral Replication in the Follicles of Lymphoid Tissues

Understanding the tissue site of viral replication will provide clues for the identification of HIV cellular reservoirs. The major replication site of HIV has been found to be in the follicular structure of lymph nodes (LNs) (16, 29, 30, 44–47). LNs are organized into cortex and paracortex areas. Cortex areas containing follicular structures consist mainly of B cells and follicular dendritic cells (FDCs), which are primarily responsible for the humoral immunity. While the paracortex areas contain predominantly T cells and conventional dendritic cells, which are primarily responsible for cellular immunity. Immunohistochemical analysis of LN tissue from patients not treated with antiretroviral drugs has revealed two distinct patterns of viral RNA staining: a diffuse staining confined within the follicles, and cell-associated staining that is scattered throughout the tissue (44). The diffused follicular presence comes from virus-immune complexes trapped on FDCs, while the cell-associated viral RNA comes from virus-infected CD4^+^ T cells (29, 43). FDCs, although not directly infected by the virus (48), can harbor the virus-immune complexes and serve as a significant reservoir, as studies have shown that this trapped virus can effectively infect CD4^+^ T cells *in vitro* even in the presence of viral neutralizing antibodies (49, 50).

While productively infected CD4^+^ T cells were found throughout the LN tissues, B cell follicles contain 31 times more infected CD4^+^ T cells than extrafollicular regions, indicating that B cell follicles are the preferred site of viral infection, replication, or both in CD4^+^ T cells (29). Upon cART, the numbers of viruses bound on FDCs and virus-infected CD4^+^ T cells are reduced dramatically (51, 52). Nevertheless, some diffused RNA (51) as well as virus-infected CD4^+^ T cells are still detected in follicles but not in extrafollicular regions (17, 51). These findings suggest that B cell follicles within LN tissues are a hotspot for viral replication in both untreated and cART-treated patients. Understanding the etiology of the B cell follicles as a replication hotspot and targeting this sanctuary site may be a feasible strategy to achieve a functional or sterilizing cure for HIV infection.

### Introduction to T_FH_ Cells

CD4^+^ T cells that reside in B cell follicles, termed T_FH_ cells, follow a distinct differentiation program that leads to a unique transcriptional profile and specialized function (13, 53). T_FH_ cells localize to B cell follicles *via* a high surface expression of CXC chemokine receptor 5 (CXCR5) and a low level of CC chemokine receptor 7 and are able to promote a humoral response due to their close proximity to B cells and FDCs (54–56). In addition to their strategic location, T_FH_ cells also express co-stimulatory receptors and cytokines that are involved in assisting B cell functioning for the humoral response (57). The upregulated genes include accessory proteins such as ICOS and SAP, which assist T–B interaction (13, 58–62), and the signature cytokine interleukin (IL)-21, which enhances the B cell response (13, 63–65). Within the CXCR5^+^ T_FH_ cells, a subpopulation that expresses the highest level of programmed death-1 (PD1) is called germinal center T_FH_ cells. These cells are able to produce the most IL-21 with the most potent B cell helper activity compared with the PD1 low counterparts (66).

The differentiation of T_FH_ cells has been an area of intensive study because of their significance in multiple human diseases (67). A complex network of key transcriptional factors that imprints the transcriptional profiles of T_FH_ cells has been identified and shown to tightly coordinate T_FH_ cell differentiation by multiple pathways (13). Cytokines, such as IL-6 (68), IL-7 (69), IL-12 (70, 71), IL-21 (72), type 1 interferons (73, 74), and transforming growth factor-β (74), have been shown to enhance T_FH_ differentiation, while IL-2 (75) inhibits differentiation by the induction of downstream signaling molecules, including signal transducer and activator of transcription proteins and Janus kinases (73, 74, 76–80). Dendritic cells (70, 81) and B cells (61, 82) are also important for T_FH_ differentiation and maintenance *in vivo* by providing antigenic stimulation, together with many of the cytokines and costimulatory signals.

## T_FH_ as a HIV Reservoir in CD4^+^ T Cells

To realize a cure for HIV infection, the major subset of CD4^+^ T cells that function as the viral reservoir must be identified and eliminated. The colocalization of HIV RNA and T_FH_ cells in B cell follicles suggests that T_FH_ cells are the major HIV reservoir within CD4^+^ T cells. Indeed, a number of studies have found that, in untreated humans and non-human primate models, T_FH_ cells harbor higher levels of viral RNA and DNA than non-T_FH_ cells (14–25). To determine whether these cells contain replication-competent virus, T_FH_ cells were sorted and activated *in vitro* and found to produce significantly more infectious virus than their non-T_FH_ counterparts (20). Importantly, in cART-treated patients who have achieved aviremia, T_FH_ cells remain the major subset with active viral transcription and produce the highest amount of replication-competent virus compared with other subsets (21, 83). Together, these findings show that T_FH_ cells are a major cellular reservoir for HIV in both untreated and cART-treated patients.

**Figure 1:**
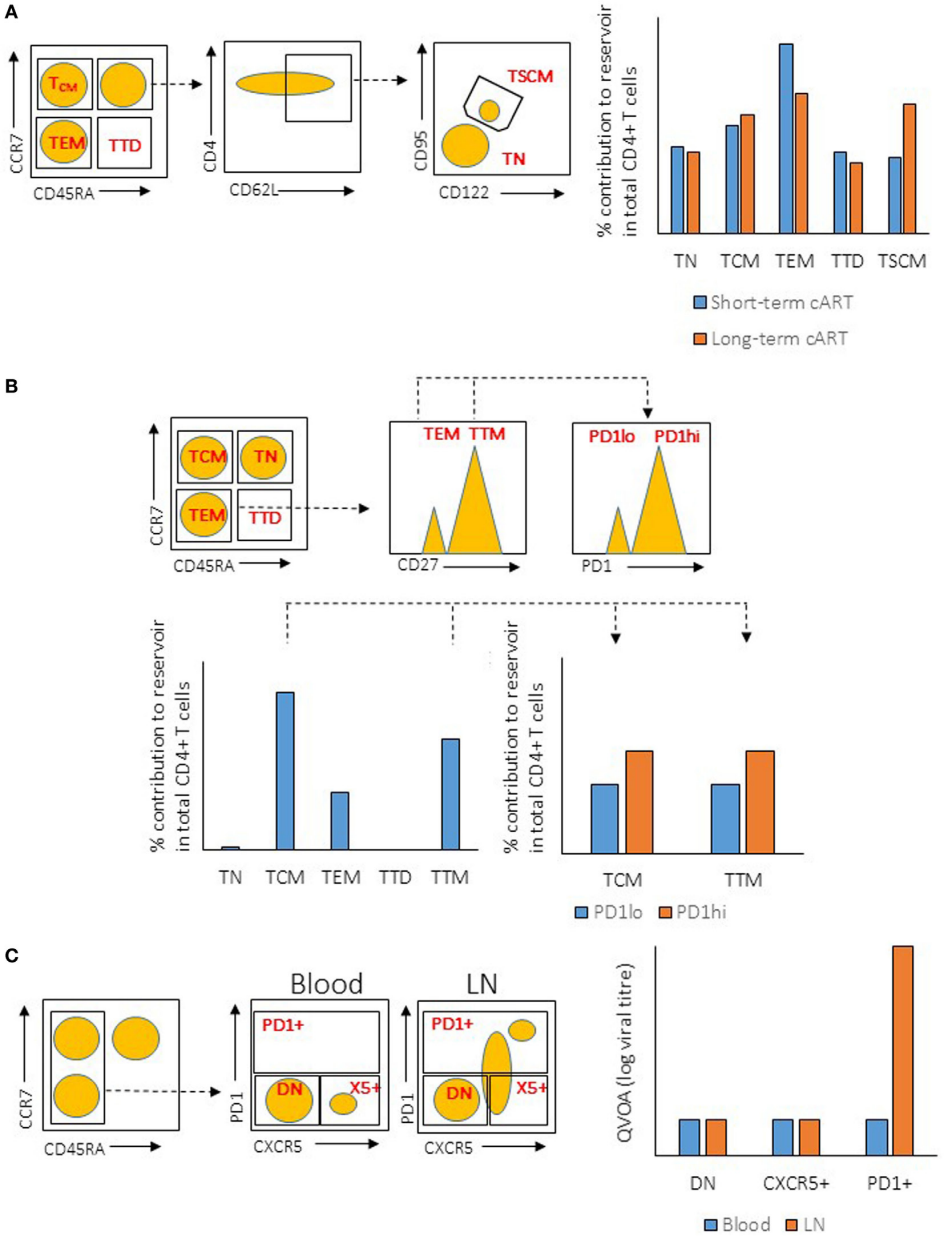
Illustration of gating strategies for CD4^+^ T cells subsets and their relative viral reservoir among different studies. **(A)** Gating strategy and CD4^+^ T cells reservoir characteristic from the study by Buzon et al. (*Left*) Plots show the gating strategy used to isolate the different population of CD4^+^ T cells from peripheral blood mononuclear cell (PBMC) (sorted population indicated in red). Dotted line indicates the subgating of the different subsets. (*Right*) Level of integrated proviral DNA from the sorted CD4^+^ T cells subsets was determined by PCR after short-term or long-term combination antiretroviral therapeutic regimen (cART). **(B)** Gating strategy and CD4^+^ T cells reservoir characteristic from the study by Chomont et al. (*Left*) Plots show the gating strategy used to isolate the different population of CD4^+^ T cells from PBMC (sorted population indicated in red). Dotted line indicates the subgating of the different subsets. (*Right*) Level of integrated proviral DNA from the sorted CD4^+^ T cells subsets was determined by PCR. **(C)** Gating strategy and CD4^+^ T cells reservoir characteristic from the study by Banga et al. (*Left*) Plots show the gating strategy used to isolate the different population of CD4^+^ T cells from PBMC or lymph node (LN) tissues (sorted population indicated in red). Dotted line indicates the subgating of the different subsets. (*Right*) ELISA for viral antigen P24 was used to quantify the level of virus production from the sorted CD4^+^ T cells subsets after stimulation by anti-CD3/CD28 antibodies [quantitative viral outgrowth assay (QVOA)]. TN, naïve T cells; TTD, terminally differentiated T cells; TEM, effector memory T cells; DN, double-negative cells.

In addition to T_FH_ cells, several other subsets of CD4^+^ T cells have been demonstrated to be preferentially infected in peripheral blood, such as central memory CD4^+^ T (T_CM_), transitional memory CD4^+^ T (T_TM_) (84), and stem cell-like memory CD4^+^ T (T_SCM_) cells (85). In these studies, CD4^+^ T cell subsets were purified using fluorophore-conjugated antibodies targeting a unique set of surface molecules that are expressed on these subsets. The purified cells were then subjected to the measurement of viral content using different assays. Although these studies identified different CD4^+^ T cell subsets as the major contributors to the HIV reservoir, the antibodies used in these studies may not have mutually excluded the different subsets of CD4^+^ T cells. For example, the antibodies and gating strategy used to identify T_CM_ and T_TM_ cells in Chomont’s studies (84) may also have included T_FH_ cells (Figure 1), which have been identified to be the major contributors for HIV reservoir in Banga et al.’s studies (83). Therefore, these studies may not necessarily be conflicting, on the contrary, the studies by Banga et al. may indeed support the early discoveries in Chomont’s studies.

On a different note, different assays have been used to assess the size of the cellular reservoir in these studies, such as PCR-based quantification of proviral DNA and quantification of cells that produce competent virus *in vitro* [namely, quantitative viral outgrowth assay (QVOA)]. Although PCR quantification is a quick, simple, and cost-effective method, that method is problematic and does not reflect the true reservoir that is responsible for viral rebound (86). The level of viral DNA in infected cells does not correlate with the production of replication-competent viruses, as the majority (approximately 98%) of viral DNA in the cells are replication defective (86–89). This could undermine studies in which viral DNA measurement is the only method used to determine the size of the reservoir, and future studies should utilize multiple quantification methods to accurately measure the true size of HIV reservoir.

**Figure 2:**
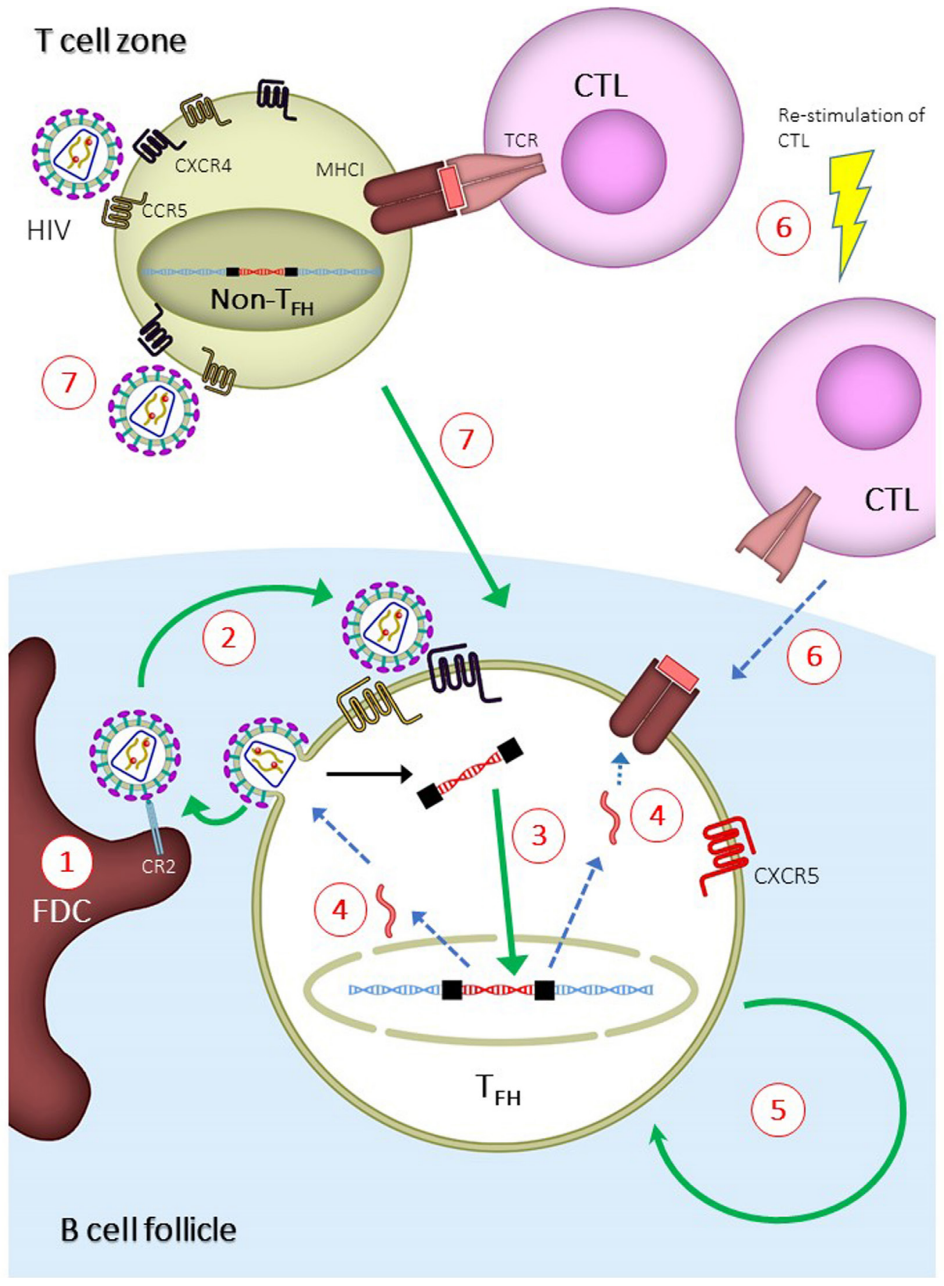
Mechanisms for the establishment of follicular T helper (T_FH_) cells as major human immunodeficiency virus (HIV) reservoir and proposed strategies to eliminate T_FH_ reservoir. Green or blue arrows indicate pathways that are enhanced or inhibited, respectively, during the establishment of T_FH_ reservoir. (1) Virus-immune complexes deposition on follicular dendritic cells (FDCs) *via* complement receptor type 2 (CR2) binding. Anti-CR2 can be used to displace virus-immune complexes. (2) Infection of T_FH_ due to suboptimal antiretroviral drug penetration, which can be overcome by development of drugs with higher potency. (3) Increased integration of viral DNA in T_FH_ cells. This can be overcome by inducing host restricting factors, such as SAMHD1, or treatment intensification with integrase inhibitor. (4) Reduced expression of viral genes, which can be overcome by latency reversal agent. (5) Long half-life and homeostatic renewal of latently infected T_FH_ cells. Long-term survival can be inhibited by targeting specific pathways, such as modulation with cytokines and auranofin. (6) Reduced infiltration of CD8^+^ T lymphocytes (CTLs) into B cell follicles. Potency and follicular infiltration of CTLs can be boosted to control T_FH_ infection. (7) Infection of non-T_FH_ and differentiation of infected non-T_FH_ to T_FH_. This can be blocked by preventing the differentiation of T_FH_ cells.

In addition to the assay method, the site of tissue sampling may also affect the conclusions in the studies. It has recently been shown that HIV replication is ongoing in tissues while it is absent in peripheral blood mononuclear cells (PBMCs) (90, 91)—a phenomenon that could stem from the insufficient penetration of cART drugs in the tissues (92). Indeed, studies have found that rebound virus originates from lymphoid tissues (47, 93), demonstrating the importance and relevance of studying the tissue viral reservoir instead of the reservoir in PBMCs. A study by Banga et al. (83) demonstrated that T_FH_ cells in LNs, but not PBMCs, are the only subset in CD4^+^ T cells that produce infectious virus after *in vitro* stimulation (or QVOA). This is a robust evidence demonstrating that T_FH_ cells are the major reservoir in long-term cART-treated patients. The ability of T_FH_ cells to produce infectious virus *in vitro* suggests that this reservoir should be eliminated or controlled in order to prevent HIV rebound. Multiple mechanisms may contribute to the establishment of T_FH_ cells as the major reservoir, and each of those mechanisms can be exploited to target and eliminate the reservoir (Figure 2). The mechanisms are discussed in the following sections.

### Virus-Immune Complexes on FDCs Facilitate T_FH_ Infection

In untreated patients, infectious virus-immune complexes displayed by FDCs, along with the close proximity to T_FH_ cells, allow B cell follicles to become a fertile ground for viral replication. Therefore, understanding the mechanism by which virus-immune complexes are loaded onto FDCs will enable the elimination of this extracellular viral reservoir, which may in turn reduce the infection of T_FH_ cells. Virus-immune complexes can form by opsonization with complement proteins (94). This is demonstrated by the trapping of virus-immune complexes on FDCs that occurs as early as 2 days after infection and before the development of an anti-HIV antibody response (95). This process is mediated by complement component 3 (C3) independent of anti-HIV antibodies (96–98). After deposition on the virus, C3 is then cleaved to iC3b and C3d, which bind to complement receptor type 2 (CR2) on FDCs (99, 100). Interestingly, during the chronic stages of the disease, B cells from untreated patients also harbor virus-immune complexes *via* CR2-binding (101). Most importantly, after 24 months of cART, by which time the viral loading on FDCs has almost fully disappeared (51, 52), FDCs isolated from these virally suppressed individuals are still able to infect CD4^+^ T cells *in vitro*, demonstrating that FDCs do contain infectious virus even under fully suppressed conditions (102). At this stage, virus is retained in the non-degrading cycling endosomes *via* binding to CR2 (100). These results demonstrate the essential role of complement and CR2 in the formation and maintenance of the extracellular viral reservoir on FDCs in B cell follicles under both before and during cART regimes. Anti-HIV antibodies generated during the later stage of the disease are able to form virus-antibody complexes and are involved in the control of the infection (103). Nevertheless, the deposition of virus-antibody complexes on FDCs has been demonstrated only in an *in vitro* study (104), and animal models are needed to examine whether this virus-antibody complex can be found on FDCs *in vivo*.

The complement system is an innate immune mechanism that induces the lysis of enveloped virus and infected cells *via* the formation of a membrane attacking complex (MAC) (105). The MAC can be activated by a classical complement pathway mediated by antibody opsonized on the targets, or an alternative pathway mediated by molecules such as complement protein C3 on the targets (105). Uninfected cells inhibit the MAC’s activity by expressing cell surface molecules that prevent the activation of the complement cascade, such as decay-accelerating factor (CD55) and membrane inhibitor of reactive lysis (CD59) (106). The activation of the MAC is therefore the balance of complement activation and inhibition, such that opsonization by complement proteins or antibodies pushes the balance toward complement activation and the induction of the MAC (106). HIV hijacks this host inhibitory system by incorporating CD55 and CD59 molecules into the viral envelope, which then inhibits the MAC-mediated lysis of the virus (107–109). This is demonstrated by the more effective MAC-mediated lysis of virus in the presence of CD59 inhibitors (94). The fact that the extracellular reservoir on FDCs has been found to contain infectious and intact virus further demonstrates that the virus evades MAC-mediated lysis. Purging this extracellular reservoir may eventually reduce the infection of T_FH_ cells, and therefore constrict the T_FH_ reservoir.

### Intrinsic T_FH_ Factors That Facilitate Viral Persistence

Note that T_FH_ cells have been found to be a major compartment for HIV production in cART-naïve patients, among whom virus production is active and *de novo* infection is prevalent. Therefore, in addition to proximity to FDCs as discussed above, two more factors may determine the level of infection in T_FH_ cells: the intrinsic susceptibility of T_FH_ cells to viral infection and the susceptibility of the infected T_FH_ cells to apoptosis.

First, T_FH_ cells isolated from peripheral blood are more permissive to HIV infection *in vitro* (15, 20, 21, 110), suggesting that intrinsic T_FH_ factors enhance the infection of T_FH_ cells. The enhanced infection could be due to a lack of intracellular host restriction factors, such as SAMHD1, allowing high degree of viral replication (110). Further studies are required to measure the expression level of other host restriction factors (111, 112) in T_FH_ cells and to determine the role of these factors in restricting viral infection. Of note, CCR5 and CXCR4—the co-receptors of HIV—are differentially expressed on T_FH_ cells that are isolated from different tissues (19, 21, 24, 67). To what degree the expression level of co-receptors or intracellular restriction factors might affect the viral infection in T_FH_ cells remains to be determined.

Second, the accumulation of highly infected T_FH_ cells in HIV patients suggests that these cells manage to evade virus-induced direct or indirect cell death. The evasion could be due to host mechanisms that “silence” the viral genome, resulting in minimal viral gene expression (113). Several mechanisms mediate the silencing of the viral genome. Specific cellular activation, such as during the resting memory state, can prevent the full expression of the viral genes because of incomplete host transcriptional machinery (113, 114). In addition, some transcription factors and epigenetic modifications have been shown to actively suppress the expression of viral genes in resting memory T cells (113–115). The transcriptional suppressor BCL6—a critical factor that initiates the T_FH_ cell differentiation program (13)—has been shown to suppress the expression of viral genes in T_FH_ cells (116). Conversely, silencing viral gene expression allows the survival of infected cells *via* two mechanisms. First, the diminished viral gene expression downregulates viral production, which in turn prevents the virus-induced cytopathic effect (117). Second, the reduced antigen presentation on MHC-I prevents recognition by CTLs (118) or natural killer cells (119) and therefore prevents cell-mediated cytotoxic killing. Both of these mechanisms contribute to reduced apoptosis and the accumulation of infected T_FH_ cells. Vigorous studies are undertaking to understand the mechanism of viral gene suppression and to determine if inducing the expression of viral genes could lead to apoptosis of the infected cells, and therefore the reduction of viral reservoir.

### Exclusion of CTLs in B Cell Follicles

CD8^+^ T lymphocytes have a well-characterized role in controlling HIV infection by inducing the death of cells harboring provirus—a feat that cannot be accomplished by cART (35, 36, 120, 121). Indeed, as mentioned above, elite controllers and long-term non-progressors have the ability to control viremia in the absence of cART (40). These patients have a strong genetic link to protective MHC-I alleles, which are the MHC-I alleles that present specific viral peptides that induce a strong anti-HIV CTL response (122). Similarly, in non-human primate models, a macaque genotype that possesses a protective MHC-I allele is often used as a model to study the role of CTLs in controlling HIV infection (123). In addition to their role in non-treated elite controllers, CTLs are also important for viral control in cART-treated subjects. This was demonstrated by a recent study in macaques showing that cART-mediated viral suppression is lost when CTLs are experimentally depleted (124). In addition to the control of active HIV infection, the non-redundant role of CTLs is also implicated in the establishment of the T_FH_ reservoir.

Another plausible hypothesis for the establishment of T_FH_ cells as a major reservoir comes from the relatively reduced prevalence of CTLs in B cell follicles. In SIV-infected elite controller macaques with a strong CTL response, the infection is confined to T_FH_ cells (16, 46). In experiments where CTLs were depleted, non-T_FH_ cells became strongly infected, while the infection in T_FH_ cells increased only marginally (16). These results demonstrate that the potent CTL response in elite controllers is able to control infection in non-T_FH_ cells, but is less able to do so in T_FH_ cells within B cell follicles (16). Immunohistochemistry confirmed the accumulation of infected T_FH_ cells in B cell follicles and, importantly, demonstrated low numbers of CD8^+^ T cells in the same anatomical location (16, 46, 125). A lack of CD8^+^ T cells in B cell follicles was also found in HIV-infected human patients (28, 50, 126). Together, these experiments show that a potent CTL response is able to eliminate the extrafollicular infection of non-T_FH_ cells but is unable to effectively control the intrafollicular infection of T_FH_ cells in both SIV-infected animal models and HIV-infected patients, probably because of the low number of CTLs in B cell follicles.

Nevertheless, our recent study has found that the CTLs in B cell follicles, although lower in number than in extrafollicular regions, have the ability to control infection in the follicles (127). Our first evidence came from the immunohistochemistry staining of LNs from untreated HIV-infected patients (127). Compared with uninfected controls, these patients have significantly increased numbers of CTLs in their B cell follicles (127). We found that, when co-stained with HIV^+^ RNA, the CTLs are juxtaposed to the infected CD4^+^ T cells in B cell follicles, indicating the ability of these follicle-infiltrating CTLs to control the infection of T_FH_ cells (127). As mentioned above, CXCR5 is the chemokine receptor expressed on T_FH_ cells that facilitates the positioning of T_FH_ in B cell follicles. Along with other groups, we found a population of CTLs expressing CXCR5 (127–132) that localizes to B cell follicles in both chronically infected mice and HIV^+^ patients (127, 129). This population—follicular cytotoxic T (T_FC_) cells—has a transcriptional profile that is distinct from non-T_FC_ CD8^+^ T cells (127–130). Notably, the T_FC_ population was found to correlate negatively with the level of viremia in untreated HIV-infected patients, demonstrating the ability of these cells to control HIV infection (129).

To determine whether T_FC_ cells are required for the control of infection in B cell follicles, we employed two mouse models of persistent viral infections: the lymphocytic choriomeningitis virus (LCMV) that infects hematopoietic cells and murid-gammaherpesvirus-4 (MuHV-4), a model virus for Epstein–Barr virus in humans who establish latent infection in B cells (127). Interestingly, we found an increased infection of T_FH_ cells compared with non-T_FH_ cells in LCMV infection, suggesting that B cell follicles are common sanctuary sites for lymphotropic virus (127). We then prevented the follicular infiltration of T_FC_ cells by deleting CXCR5 expression in CTLs and found a further accumulation of infected T_FH_ cells (127). A similar accumulation of infected B cells was found in MuHV-4 infection in the absence of CXCR5 on T_FC_ cells (127). Together, these results demonstrate that T_FC_ cells, although low in quantity, are able to control infection in B cell follicles.

The ability of T_FC_ cells to control HIV/SIV infections is further demonstrated in recent studies (128, 131). In these studies, CXCR5^+^ CD8^+^ T cells from SIV-infected macaques (131) and HIV-infected patients (128) were found to eliminate virus-infected T_FH_ cells when cocultured *in vitro*. Moreover, the anti-HIV activity of CXCR5^+^ CD8^+^ T cells from HIV-infected patients was significantly enhanced by a bispecific antibody (128). The bispecific antibody is a fusion antibody that contains two covalently linked monoclonal antibodies (mAbs): an anti-CD3 mAb that activates polyclonal CTLs and an anti-HIV mAb (VRC07) that recognizes viral antigen on infected cells (128). The use of the bispecific antibody is a clever design as majority of the CXCR5^+^ CD8^+^ T cells in the follicles were found to be non-HIV specific (128), and with the use of this antibody, one can “borrow” the cytotoxicity of these polyclonal CXCR5^+^ CD8^+^ T cells to eliminate T_FH_ infection in B cell follicles. All in all, the discovery of T_FC_ cells in chronic infections and their ability to control infection in follicles are important steps toward understanding the follicle sanctuary for HIV infection, and devising strategies to eliminate infection by boosting the number and cytotoxicity of T_FC_ cells.

### Long-term Survival of T_FH_ Cells during cART

Combination antiretroviral therapeutic regimen suppresses viral replication, but cannot eliminating cells with integrated provirus. While the provirus-harboring cells evade viral cytopathic effect and CTL-mediated cell death, these infected cells can persist in the body *via* homeostatic proliferation due to the ability of the integrated provirus to propagate *via* mitotic division. The steady maintenance of this viral reservoir is therefore dependent on the infected cells’ rate of homeostatic proliferation and susceptibility to apoptosis, and the duration of these cells to remain in the interphase of the cell-division cycle (133). Indeed, the half-life of virus-infected resting memory T cells during cART is approximately 44 months, reflecting the long-term homeostatic maintenance of memory T cells (134, 135). Among the resting memory T cells, infected T_CM_ and T_TM_ cells persist *via* T cell receptor-driven and IL-7-mediated homeostatic proliferation, respectively (84). T_FH_ cells, as described above, contain more infected cells than T_CM_ and T_TM_ cell populations, constituting the major reservoir within memory T cells during cART (21, 83). While the half-life of infected T_FH_ cells during cART is not known, T_FH_ cells are able to form long-term memory (136–138) and require IL-7 signaling to maintain cell numbers *in vivo* (68, 139), indicating a mechanism similar to T_TM_ cells for the persistence of the viral reservoir. T_FH_ cells from HIV-infected patients were found to express higher levels of an anti-apoptotic gene, BCL2, which may further facilitate the persistence of the T_FH_ reservoir by suppressing apoptosis (140). In addition to maintaining cell survival and reducing apoptosis, T_FH_ cells also appear to be preferentially multiplied compared with their non-T_FH_ counterparts (17, 20, 22, 24, 141). The expansion of T_FH_ cells is positively correlated with the level of IL-6, a cytokine that promotes T_FH_ differentiation in the plasma (7, 17, 22). The molecular mechanisms for the long-term persistence and expansion of T_FH_ cells remain poorly understood; however, targeting these pathways is a plausible strategy to reduce the viral reservoir in this compartment.

## How to Eliminate the T_FH_ Reservoir in B Cell Follicles

In this review, we define the HIV reservoir as the cellular populations that are able to generate infectious virus after treatment interruption. According to this definition, the HIV reservoir would encompass the reservoirs with the following characteristics: (1) latently infected cells with integrated proviruses that do not express viral genes during ART, yet are able to generate infectious virus after treatment interruption. This reservoir includes cells that contain HIV DNA but not HIV RNA (142); (2) infected cells with integrated proviruses expressing viral genes, which contain both cell-associated HIV DNA and RNA (142); (3) active viral replication in tissues reservoir, which is identified by the evolution of HIV DNA at tissue sanctuary sites (see below); (4) extracellular viral reservoir, which consist of infectious viruses bound to FDCs in B cell follicles. All of the abovementioned reservoirs are either found in T_FH_ cells (point 1 and 2) or are related to tissues’ sanctuary sites (point 3) and B cell follicles (point 4). Therefore, targeting the B cell follicles in lymphoid tissues might significantly impact all types of reservoirs. In this section, we discuss the potential strategies for targeting the B cell follicles to eliminate the HIV reservoir (Figure 2).

### Eliminating Residual Replication and Purging the FDC Reservoir

As discussed above, FDCs may facilitate the infection of T_FH_ cells due to the proximity of the two within B cell follicles. Nonetheless, it is disputable whether active viral infection and replication occur *in vivo* under cART that blocks the *de novo* infection of CD4^+^ T cells [see the review by Eisele and Siliciano (143)]. Residual viremia after prolonged cART has been detected at below 50 copies/mL using sensitive methods, indicating a continuous production of virus. The source of this viremia—whether it is produced from infected cells with integrated provirus or is due to continual viral replication that escapes cART suppression—is still debatable. Some studies have shown conclusive evidence of continual viral replication in lymphoid tissues (90, 92), while contradictory evidence was observed in PBMCs (144–147). For lymphoid tissues, insufficient penetration of antiretroviral agents into the lymphoid follicles could be one of the mechanisms that cause residual viral replication (92). For PBMCs, a definitive study on Raltegravir, an antiretroviral drug that inhibits the integration of linear cDNA into chromosomes, showed that viral replication is continual in the PBMCs of some cART patients (148, 149). During active viral infection, the reversed transcribed linear cDNA must be integrated into the chromosome to allow the production of new virus. At this stage, the host DNA repair mechanisms are able to induce the self-ligation of linear DNA to form circular 2-long terminal repeat (2-LTR) DNA as a way of preventing chromosomal integration (148, 149). After Raltegravir administration to cART-treated patients, the level of 2-LTR increased in 29% of the individuals, suggesting that active viral replication and infection continue to occur in these individuals (149). This is likely to be due to the suboptimal suppression of viral replication by standard cART.

In summary, patients enrolled for reservoir purging trials should have minimal residual viral replication in the body in order to prevent the replenishment of the reservoir after purging. FDCs, on the other hand, can store virus-immune complexes that facilitate the reinfection of T_FH_ cells in B cell follicles. Virus-immune complexes can be displaced from FDCs and B cells using anti-CR2 antibodies (150), or directly destroyed by inducing the formation of a MAC using anti-CD55 antibody or the bacterial toxin intermedilysin that blocks host inhibitor CD55 (94). Minimizing the residual replication and loading of the virus-immune complex on FDCs would be an important consideration to prevent the accumulation of infected T_FH_ cells before attempting to purge the provirus-containing T_FH_ cells.

### Reduce the Number of Memory T_FH_ Cells

The long half-life of resting memory T cells enables the persistence of the HIV reservoir. Purging total memory cells, including memory T_FH_ cells, could be a plausible method of reducing the T_FH_ reservoir. A proof of concept for this strategy is the use of auranofin, a gold-based chemical that targets the metabolic profile of T_CM_ and T_TM_ cells (151) to reduce the size of the latent reservoir (152). In combination with cART, auranofin has been found to reduce viral rebound and spontaneously control SIV in macaques (153), which resemble human PIVCs. A similar strategy can be used to target T_FH_ cells, as T_FH_ cells have been shown to possess a metabolic profile different from other CD4^+^ T subtypes (57, 154–156). Other survival pathways can also be targeted, such as by blocking the activity of IL-7, which maintains the long-term survival of T_FH_ cells (69), as well as by blocking the activity of the antiapoptotic BCL2 gene that is highly expressed on T_FH_ cells (140). Similarly, modulating the differentiation of T_FH_ cells may also reduce the total number of T_FH_ cells. We have shown that low-dose IL-2 treatment is able to reduce the number of T_FH_ cells in patients with systemic lupus erythematosus (157). Treatment with IL-2 or anti-IL-6 may inhibit T_FH_ cell differentiation and prevent the accumulation of infected T_FH_ cells. Other novel reagents can also be developed to target the complex regulation of T_FH_ differentiation (158). However, it is important to acknowledge that a gross reduction of T_FH_ cells may negatively impact the humoral immune response, including the anti-HIV antibody response that might have been established over the course of infection. Therefore, more specific targeting of latently infected T_FH_ cells instead of total T_FH_ reduction may be a superior strategy.

### Specific Targeting of Latently Infected T_FH_ Cells: Shock and Kill

A new method, “shock and kill,” has been proposed for the elimination of the latent HIV reservoir (159). As mentioned above, silencing viral gene expression prevents the presentation of viral peptides on MHC-I and their recognition by CTLs. To overcome this hurdle, it was proposed to induce the expression of viral genes, which could in turn expose latently infected cells to virus-induced cytopathic effect and immune system-mediated killing (160). In a clinical trial in which cART patients were treated with a histone deacetylase inhibitor—an epigenetic modifier drug belonging to a group of latency reversal agents (LRAs)—viral gene expression was induced in latently infected cells in cART-treated patients (160, 161). Nevertheless, even with the increased viral transcription, treatment with LRAs *in vivo* did not reduce the size of the viral reservoir (160), and viral rebound was not affected after cART regime was interrupted (161). This observation suggests that “shocking” the latently infected cells to express viral gene products alone is not sufficient, and that the simultaneous induction of the immune system to “kill” is also required (159, 162). To that end, the enhancement of CTL function may be the most suitable candidate in this strategy to kill the latently infected cells (159, 162).

Cytotoxic killing by CTLs is needed to control and eliminate the latent cellular reservoir; however, CTLs in HIV-infected patients are often defective due to exhaustion (163) and mutations in viral genes that allow infected cells to escape elimination by CTLs (164). Exhaustion is a state of CTLs that is characterized by reduced effector functions and failure to control infections, which is typically observed in chronic infections such as with HIV and hepatitis C and B viruses (163). Persistent antigenic signaling and inflammatory signals contribute to the exhaustion of CTLs, and revitalizing them has been shown to restore immunity (163). Indeed, *in vitro* LRAs showed that latently infected CD4^+^ T cells can only be killed by IL-2-activated allogenic CTLs, but not by unstimulated CTLs, demonstrating that the restimulation of exhausted CTLs can be useful to eliminate the latent reservoir (162). In addition to CTL exhaustion, viral CTL escape mutations also play a significant role in viral persistence (164). In patients treated early with cART, CTL clones are able to recognize non-escaped mutations and kill infected CD4^+^ T cells (165). In patients who did not receive treatment early, CTLs failed to recognize virus-infected cells due to emergence of escape mutations (165). These studies demonstrate that revitalizing CTL clones in addition to targeting non-escape mutations are pre-requisites for the shock-and-kill strategy.

Finally, to target the T_FH_ cell reservoir, CTLs need to penetrate the B cell follicular sanctuary to reach the latently infected T_FH_ cells. The depletion of B cell follicles using depleting antibodies has been proposed to achieve this. However, depleting B cell follicles can result in a dysfunctional humoral response and a compromised anti-HIV antibody response. To specifically target infected T_FH_ cells, promoting the infiltration of CTLs into B cell follicles is a preferred strategy. Recent publications highlighted the critical role of CXCR5^+^ T_FC_ cells in the control of follicular infections (127–129, 131), suggesting possible therapeutic benefits of boosting the number and antiviral activity of T_FC_ cells in B cell follicles. Finally, to enable CTLs’ detection of infected T_FH_ cells, special consideration might be needed to “shock” viral gene expression in T_FH_ cells due to the ability of the T_FH_-specific transcription factor BCL6 to suppress viral gene expression.

All in all, multiple strategies are needed to reduce and control the viral reservoir in T_FH_ cells. This may eventually bring the reservoir under control to achieve a functional cure, which may in turn eliminate viral rebound and viral transmission in the absence of cART.

## Conclusion

This review originates from the recent discoveries demonstrating that T_FH_ cells are the major viral reservoir among CD4^+^ T cells. We have proposed hypotheses to explain the formation of this reservoir and strategies for its elimination. The major HIV reservoir in T_FH_ cells is a concrete observation that agrees well with the long-observed hotspot for HIV replication, the B cell follicles. Nonetheless, more studies are needed to validate the significance of this cellular reservoir and to determine whether removing the reservoir can considerably delay or eliminate viral rebound. While viral DNA and RNA in CD4^+^ T cells have been the strongest predictor of the “time to viral rebound” [that is, higher levels of cell-associated RNA and DNA lead to a shorter time to viral rebound after treatment is interrupted (166)], it would be interesting to learn whether the level of provirus in T_FH_ cells can be used as a superior predictor for the time to rebound. Hence, in addition to helping us understand HIV pathogenesis, the T_FH_ reservoir can also be developed as a biomarker to assess the efficacy of therapeutic interventions in HIV cure studies.

At least two types of reservoir exist in B cell follicles during cART: the virus-immune complexes retained on the surface of antigen presenting cells and the intracellular proviral reservoir that produces infectious virus when treatment is interrupted. Both of these reservoirs should be eliminated to prevent viral rebound, and extra effort is needed to target these reservoirs in B cell follicles. The hypotheses and strategies proposed here ought to be empirically verified in animal models to determine the true cause(s) for the establishment of the T_FH_ reservoir and to determine the most rational and promising therapeutic interventions. After some great successes but also some limitations in using SIV-infected non-human primate models to study human HIV infection, humanized mice are also being developed as excellent tools for the study of HIV pathogenesis. The successful grafting of T_FH_ cells into the mice would allow us to study the role of the T_FH_ cell reservoir in chronic HIV disease (24). The role of T_FH_ cells as the major reservoir among CD4^+^ T cells warrants further investigation into its role in HIV pathogenesis, and such crucial findings should not be overlooked in the development of therapeutic strategies to cure HIV infection.

## Author Contributions

YL and DY wrote the manuscript. AA revised the manuscript.

## Conflict of Interest Statement

The authors declare that the research was conducted in the absence of any commercial or financial relationships that could be construed as a potential conflict of interest.
